# Association between parasport participation and hand function in wheelchair users with chronic spinal cord disorders: a cross-sectional pilot study

**DOI:** 10.1186/s13102-025-01378-x

**Published:** 2025-12-05

**Authors:** Hiroshi Yuine, Hirotaka Mutsuzaki, Ryoko Takeuchi, Yuichi Yoshii, Yukiyo Shimizu, Taku Yasuda, Kazushi Hotta, Hideki Shiraishi, Kaori Tachibana

**Affiliations:** 1 Department of Occupational Therapy, School of Health Sciences, Ibaraki Prefectural University of Health Sciences, 4669-2 Ami, Ami-machi, Inashiki-gun, Ami, Ibaraki 300-0394 Japan; 2 Center for Medical Sciences, Ibaraki Prefectural University of Health Sciences, Ami, Ibaraki 300-0394 Japan; 3 Department of Orthopedic Surgery, Ibaraki Prefectural University of Health Sciences Hospital, Ami, Ibaraki 300-0331 Japan; 4 Department of Orthopaedic Surgery, Tokyo Medical University Ibaraki Medical Center, Ami, Ibaraki 300-0395 Japan; 5 Department of Rehabilitation Medicine, Faculty of Medicine, University of Tsukuba, Tsukuba, Ibaraki 305-8575 Japan; 6 Department of Physical Therapy, School of Health Sciences, Ibaraki Prefectural University of Health Sciences, Ami, Ibaraki 300-0394 Japan

**Keywords:** Hand function, Joint instability, Parasport, Spinal cord disorders, Wheelchair users

## Abstract

**Background:**

Wheelchair users with chronic spinal cord disorders who participate in parasports reportedly experience more severe chronic wrist pain or triangular fibrocartilage complex injuries. Their activities include parasport participation and wheelchair use in daily life. Hence, clarifying the association between parasport participation on hand function and distal radioulnar joint (DRUJ) instability is essential to avoid overestimation or underestimation of the involved risk. In this study, we aimed to investigate the association between parasport participation and hand function as well as DRUJ instability in wheelchair users with chronic spinal cord disorders.

**Methods:**

Wrists of wheelchair users with chronic spinal cord disorders were evaluated using force-monitor ultrasonography. Hand function was evaluated by measuring the range of motion (ROM) and muscle strength for wrist flexion, extension, radial and ulnar deviation, as well as grip strength, arm and forearm circumference, and DRUJ instability using the ballottement test. The Quick Disability of Arm, Shoulder, and Hand was used to assess difficulties and pain experienced during daily life activities and parasport participation.

**Results:**

Overall, 30 wrists of 15 wheelchair users (mean age, 58.3 years; range, 33–76 years) were evaluated. Neither DRUJ instability nor hand function, including ROM and muscle strength, significantly differed between the parasport participants (*n* = 6; 12 wrists) and non-participants (*n* = 9; 18 wrists).

**Conclusions:**

Among the small number of wheelchair users with chronic spinal cord disorders investigated in the present study, no association was observed between parasport participation and either DRUJ instability or hand function. These findings suggest that recreational parasport participation may not adversely affect hand function in wheelchair users.

**Trial registration:**

The protocol was registered with the University hospital Medical Information Network (UMIN) Clinical Trials Registry (UMIN000043343) [Date of first registration: 02/16/2021].

## Background

Parasports are not only practiced by athletes but also by non-athletes seeking to improve physical and mental function, social participation, and achieve personal goals [1]. Most individuals with spinal cord disorders experience traumatic injuries, and some of these individuals commence parasport participation during rehabilitation [2]. Participation in parasports offers several benefits to individuals with spinal cord disorders, including improvements in physical and mental function, socialization, and psychological aspects. However, medical complications are a barrier to participation [2]. Therefore, injury prevention among participants is important for ensuring continued participation in parasport as a meaningful and purposeful activity.

A systematic review summarized the incidence of injuries by sport, and various parasport-related health problems have been reported [3]. Most studies investigating injuries associated with parasports involved elite athletes [4–6], and only a few studies included non-athletes [1, 7]. Parasport participants often experience shoulder, wrist, and elbow injuries, regardless of whether they are athletes or non-athletes [1, 6, 7]. Meanwhile, the effectiveness of injury prevention measures has only been verified for shoulder injuries [8–11]. Investigating the impact of parasport participation or non-participation on upper limb function, particularly involving the wrist, is necessary for developing preventive measures.

One of the most important injuries that need to be considered among wheelchair parasport participants is triangular fibrocartilage complex (TFCC) injury, and athletes have more severe injuries compared to non-athletes [12]. A survey of wheelchair basketball athletes found that 38.9% of participants had TFCC injuries [13]. Moreover, injuries to the TFCC have been reported among wheelchair users during daily activities [14]. Distal radioulnar joint (DRUJ) instability due to TFCC injury reportedly reduces hand function, including forearm rotation torque [15]. Reduced hand function impacts parasport participation; however, the relationship between DRUJ instability and hand function in non-athlete parasport participants remains unclear.

Parasport participants with shoulder, elbow, and wrist joint injuries often include those with spinal cord disorders [7]. Parasport participation and wheelchair use in daily life are relevant not only to athletes but also to non-athletes, particularly among individuals with spinal cord disorders. Underestimating changes in hand function may increase the risk of disability, while overestimating them may discourage continued participation in parasports. Therefore, clarifying the association between parasport participation and hand function in non-athlete wheelchair users with spinal cord disorders is essential to avoid misjudging these risks. We hypothesized that parasport participants who use wheelchairs have limited hand function, compared with non-participants. Therefore, we aimed to investigate the association of parasport participation with hand function and DRUJ instability in wheelchair users with spinal cord injury.

## Methods

This cross-sectional pilot study was approved by the institutional review board of Ibaraki Prefectural University of Health Sciences (e306). The study protocol was entered into the University hospital Medical Information Network (UMIN) Clinical Trials Registry (UMIN000043343) before participant enrollment commenced [Date of first registration: 02/16/2021]. Study participants were recruited through posters displayed in the hospital, as well as through direct referrals from physicians and rehabilitation staff. The nature of the study and the data to be collected were explained in writing and verbally to all participants in accordance with the Declaration of Helsinki. Informed consent was obtained from them via a consent form.

### Participants

The participants in this study were chronic wheelchair users who were outpatients. The inclusion criteria required participants to be wheelchair users, with no restrictions based on age, diagnosis, or previous wheelchair use experience. The exclusion criteria were as follows: (1) acute deterioration of condition, (2) obvious cognitive decline, and (3) determination of ineligibility for study participation by the attending physicians. Demographic (age, sex, and dominant hand) and medical (disease name, history, and duration since injury) data were obtained from medical records and questionnaires. Participants were assessed for parasport participation, DRUJ instability, and hand function.

### Assessment of DRUJ instability using ultrasonography

**Fig. 1:**
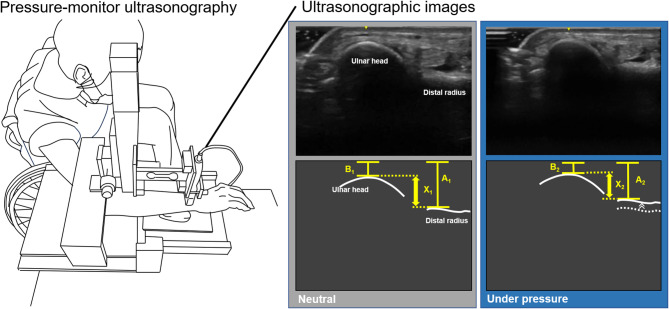
Evaluation of distal radioulnar joint instability X (amount of displacement) = X_1_ - X_2_ X_1_ (distance between the radius and the ulna at neutral position) = A_1_ - B_1_ X_2_ (distance between the radius and the ulna under pressure position) = A_2_ - B_2_ A = distance to the dorsal surface of the distal radius B = distance to the dorsal surface of the ulna head

Participants’ wrists were evaluated for DRUJ instability using force-monitor ultrasonography (Fig. 1) [16]. Force-monitor ultrasonography was designed to assess applied force during cyclic compression of the ulnar head [17]. The force applied to the wrist was measured using a strain gauge sensor at a frequency of 200 Hz. The apparatus was set at 3.0 mm and 1.5 Hz for vertical downward compression of the transducer, based on settings shown to have high intra- and inter-rater reliability in a previous study [18]. The evaluation procedure was as follows: (1) While seated in a wheelchair, the participant placed their forearm on the device’s support stand in a pronated position. (2) The forearm was adjusted so that the distal radius and pisiform were supported on the palmar side. (3) The ultrasound probe was positioned to contact the ulnar head from the dorsal side using ultrasound gel. (4) The forearm’s long axis position was adjusted to visualize the center of the ulnar head and the distal dorsal radius on the ultrasound image. (5) The lateral position was set so that the ulnar head was centered in the ultrasound image along the forearm’s long axis. (6) The vertical position was adjusted to ensure the probe was in contact with the dorsal side of the ulnar head. (7) The starting position was defined as when pressure data were reset to zero and mechanical compression was applied. (8) Ultrasound recorded the radius and ulna movement, while a force sensor simultaneously measured pressure applied to the DRUJ. Radioulnar displacement (X mm) and applied force (N) were measured five times, with the mean values used for analysis. The displacement-to-force ratio (mm/N), normalized by the applied force, served as an indicator of DRUJ instability.

### Assessment of hand function

Participants’ hand function was assessed, including right and left grip strength, wrist strength (flexion, extension, radial deviation, ulnar deviation), range of motion (ROM; flexion, extension, radial deviation, ulnar deviation), upper arm circumference, forearm circumference, presence of DRUJ instability using the ballottement test, fovea sign, and spontaneous pain. In the ballottement test, instability was determined as positive or negative based on the examiner’s subjective evaluation of manual resistance. The Quick Disability of the Arm, Shoulder, and Hand (Q-DASH) was used to assess function and pain in daily life, work, and sports activities. Participants were asked to detail their sports history [19].

Grip strength was evaluated using a grip strength meter (T.K.K. 5101; Takei Electric Industries, Tokyo, Japan), and the average of two measurements was used for data analysis. A hand-held dynamometer (Micro FET2; Hoggan Scientific, LLC, Salt Lake City, UT, USA) was used to evaluate hand joint muscle strength, measuring muscle strength against manual resistance by the therapist in each direction of motion: flexion, extension, radial deviation, and ulnar deviation. ROM was evaluated using a goniometer (GS-100; OG Wellness, Tokyo, Japan), and passive ROM was recorded in flexion, extension, radial deviation, and ulnar deviation. Upper arm and forearm circumferences were evaluated using a tape measure (GS11-004; OG Wellness). The circumferences were measured at the point of maximum girth. Manual examination of the wrist joints included DRUJ instability findings obtained using the ballottement test, and fovea sign and spontaneous pain were evaluated for wrist-related ulnar pain. These hand function assessments were performed after participants reported pain and difficulty related to the upper extremity, including the hand and wrist, using the Q-DASH. Scores were calculated for Q-DASH function/pain, work, and sports. Additionally, participants were asked concerning their parasport participation in relation to their Q-DASH sports scores. The Q-DASH scores were converted to a maximum of 100 points, with higher scores indicating greater difficulty in each category. A single occupational therapist (HY) performed these hand function assessments.

### Statistical analysis

The pilot study did not require a formal power calculation for sample size estimation; instead, the minimum sample size for each group was set at 12 wrist joints based on a recent review [20]. The parameters of each group were tested for normality using the Shapiro–Wilk test. The Mann–Whitney U-test was used to compare parameters between the parasport participants and non-participants. The chi-square test was applied to compare the DRUJ ballottement test, fovea sign, and spontaneous ulnar pain between parasport participants and non-participants. All statistical analyses were conducted using SPSS Statistics, version 27 (IBM Corp., Armonk, NY, USA). Statistical significance was set at *P* < 0.05. Data are presented as means ± standard deviations or medians (interquartile range, IQR), and P-values and effect sizes (r or φ) were calculated. The effect size r was calculated based on the Z-score and sample size (n) using the following equation: effect size r = Z score/√n. The effect size r and φ value were interpreted as follows: r or φ value >0.10 indicated a small effect; >0.30, medium; and >0.50, large [21]. The effect size d was converted to r using the following formula: effect size d = 2r/√(1-r^2^) [22]. Based on the calculated effect size and a confidence level of 95% (α = 0.05), power (1-β) was calculated using a post-hoc power analysis. A power (1-β) of 0.80 or less was interpreted as a high risk of Type II error [21]. Power analysis was performed using G*Power v3.1.9.7 (University of Dusseldorf, Dusseldorf, Germany).

## Results

Fifteen wheelchair users with chronic spinal cord disorders (mean age, 58.3 ± 12.3 years; age range, 33–76 years; mean onset age, 20.3 ± 10.3 years; onset age range, 7–52 years) were evaluated (Table 1). Overall, 12 hands of six participants with parasport experience and 18 hands of nine participants without parasport experience were evaluated. The parasports included boccia (*n* = 2), wheelchair basketball (*n* = 2), takkyu volley (*n* = 1), trike (*n* = 1), handcycle (*n* = 1), wheelchair marathon (*n* = 1), wheelchair table tennis (*n* = 1), and para powerlifting (*n* = 1) (multiple answers included). In the parasport group, only one participant had athlete-level experience, such as participation in international games, and the other five participants were all at the recreational level. The parasport group included participants with a history of TFCC injury (*n* = 1), scaphoid fracture (*n* = 1), rotator cuff injury (*n* = 1), and femoral neck fracture (*n* = 1). The non-parasport group included participants with a history of TFCC injury (*n* = 4); thumb carpometacarpal joint disorder and lateral epicondylitis (*n* = 1); carpal tunnel syndrome, shoulder inflammation, elbow joint pain, and mallet finger (*n* = 1); and femoral neck fracture, joint pain in both hands, and cervical and lumbar hernias (*n* = 1).

**Table 1:** Demographic characteristics of the parasport participants and non-participants

Group	Parasport participants (*n* = 6)		Non-participants (*n* = 9)		*P*-value	Effect size	Power (1-β)
	Mean ± SD	Median (IQR)	Mean ± SD	Median (IQR)			
Level of injury (number)	Thoracic (4) Lumbar (2)		Thoracic (7) Lumbar (2)				
Athletic level (number)	Athlete level (1) Recreational level (5)						
Age (years)	56.8 ± 6.3	57.5 (51.0–62.0)	59.3 ± 15.4	65.0 (47.0–74.0)	0.53 **(n.s.)**	*r* = 0.18 (small)	0.10
Sex (number)	Male (4)	Female (2)	Male (7)	Female (2)	1.00 **(n.s.)**	φ = 0.12 (small)	0.08
Body weight (kg)	66.0 ± 16.4	65.9 (56.3–75.7)	70.6 ± 12.2	68.2 (61.2–81.1)	0.78 **(n.s.)**	*r* = −0.09 (none)	0.06
Onset time (years)	26.0 ± 13.3	23.5 (17.3–31.0)	16.4 ± 5.9	16.0 (11.0–22.0)	0.07 **(n.s.)**	*r* = −0.49 (medium)	0.48
Quick DASH (score)	4.73 ± 4.02	4.55 (1.70–6.53)	9.07 ± 8.28	6.82 (1.14–18.2)	0.46 **(n.s.)**	*r* = −0.22 (small)	0.12

*n.s.* not significant (*p* > 0.05), *SD* standard deviation, *IQR* interquartile range, *DASH* Disability of Arm, Shoulder and Hand

Each of the DRUJ instability and hand function items included both normal and non-normal distributions. Neither DRUJ instability nor hand function, including ROM and muscle strength, significantly differed between the parasport and non-parasport groups (Table 2). The effect size was “small” or “none” for all hand function measures, and the statistical power was insufficient.

**Table 2:** Comparison between parasport participants and non-participants

Group	Parasport participants (*n* = 6; 12 wrists)		Non-participants (*n* = 9; 18 wrists)		*P*-value	Effect size	Power (1-β)
	Mean ± SD	Median (IQR)	Mean ± SD	Median (IQR)			
Displacement (mm)	0.47 ± 0.24	0.41 (0.27–0.70)	0.56 ± 0.28	0.50 (0.37–0.70)	0.37 **(n.s.)**	*r* = 0.17 (small)	0.14
Applied force (N)	4.27 ± 0.71	4.25 (3.62–4.96)	4.58 ± 0.68	4.50 (4.15–5.16)	0.22 **(n.s.)**	*r* = 0.23 (small)	0.22
Displacement-to-force ratio (mm/N)	0.11 ± 0.06	0.10 (0.06–0.17)	0.13 ± 0.09	0.10 (0.09–0.16)	0.57 **(n.s.)**	*r* = 0.11 (small)	0.09
Grip strength (N)	317.0 ± 76.4	322.6 (233.6–385.9)	308.3 ± 89.9	306.2 (246.3–368.2)	0.60 **(n.s.)**	*r* = 0.10 (small)	0.08
Muscle strength (N)							
Flexion	64.2 ± 13.3	63.3 (54.9–69.6)	60.0 ± 12.3	57.4 (49.5–73.1)	0.52 **(n.s.)**	*r* = 0.12 (small)	0.10
Extension	63.8 ± 14.5	65.7 (51.5–76.5)	60.3 ± 15.3	59.3 (45.8–70.6)	0.35 **(n.s.)**	*r* = 0.17 (small)	0.15
Radial deviation	52.8 ± 12.3	52.0 (43.6–65.5)	52.9 ± 9.7	54.4 (42.9–59.6)	0.98 **(n.s.)**	*r* = 0.004 (none)	0.05
Ulnar deviation	54.7 ± 9.8	56.9 (48.8–60.8)	54.6 ± 12.5	51.5 (46.6–63.0)	0.72 **(n.s.)**	*r* = 0.07 (none)	0.06
ROM (°)							
Flexion	65.0 ± 9.8	67.5 (61.3–70.0)	62.5 ± 9.6	65.0 (53.8–70.0)	0.37 **(n.s.)**	*r* = 0.17 (small)	0.14
Extension	70.0 ± 12.2	72.5 (65.0–80.0)	66.7 ± 9.4	67.5 (60.0–75.0)	0.29 **(n.s.)**	*r* = 0.20 (small)	0.18
Radial deviation	22.1 ± 4.5	22.5 (20.0–25.0)	21.7 ± 6.2	20.0 (20.0–26.3)	0.88 **(n.s.)**	*r* = 0.03 (none)	0.05
Ulnar deviation	40.0 ± 8.8	40.0 (36.3–48.8)	41.1 ± 5.6	40.0 (40.0–45.0)	0.85 **(n.s.)**	*r* = 0.04 (none)	0.05
Upper arm circumference (cm)	31.7 ± 3.3	32.0 (28.6–35.0)	32.2 ± 3.0	31.5 (30.5–33.6)	0.82 **(n.s.)**	*r* = 0.04 (none)	0.82
Forearm circumference (cm)	27.0 ± 2.6	27.5 (24.6–28.9)	28.1 ± 2.7	27.8 (26.0–29.5)	0.35 **(n.s.)**	*r* = 0.18 (small)	0.35
Ballottement test (wrists)	− (12)	+ (0)	− (16)	+ (2)	0.50 **(n.s.)**	φ = 0.22 (small)	0.23
Fovea sign (wrists)	− (12)	+ (0)	− (17)	+ (1)	1.00 **(n.s.)**	φ = 0.15 (small)	0.13
Spontaneous ulnar pain (wrists)	− (12)	+ (0)	− (17)	+ (1)	1.00 **(n.s.)**	φ = 0.15 (small)	0.13

*n.s.* not significant (*p* > 0.05), *SD* standard deviation, *IQR* interquartile range, *DASH* Disability of Arm, Shoulder and Hand, *ROM* range of motion, displacement, radioulnar displacement, applied force, force applied to the wrist, displacement-to-force ratio, displacement/applied force ratio

## Discussion

This pilot study focused on wheelchair users with chronic spinal cord disorders and investigated the relationship between DRUJ instability and hand function depending on parasport participation. No significant differences in DRUJ instability and hand function were observed between parasport participants, mainly at recreational level, and non-participants. However, post-hoc power analysis revealed that the power was 0.80 or less for all measures except forearm circumference (1-β = 0.82), suggesting that statistically significant differences may have gone undetected. These findings indicate that a true effect might exist but was not detected due to limited statistical power, highlighting a potential risk of Type II error.

To the best of our knowledge, no previous study has quantitatively evaluated and analyzed DRUJ instability in non-athlete patients with chronic spinal cord disorders. Clinical evaluation of DRUJ instability is typically conducted using manual stress tests, such as the ballottement test and fovea sign [23, 24]. These are subjective assessments that cannot be quantified or rigorously analyzed, making evaluations of DRUJ instability challenging. Thus, efforts have been made to quantify DRUJ instability using various imaging tests, including computed tomography and ultrasound [24]. Force-monitor ultrasonography can quantitatively evaluate DRUJ instability while posing a minimal burden on the patient, and its reliability and validity have been verified [18, 25]. Reports have indicated changes in DRUJ stability in healthy individuals due to aging and sex using this method, and some studies have captured changes over time due to treatment [26, 27]. For wheelchair basketball athletes, evaluation was possible while they were in their wheelchairs [13]. The parasports, in which the participants engaged included sports that placed repeated stress on the wrist, such as wheelchair basketball, handcycle, and wheelchair marathon. However, no significant differences were observed in non-athlete wheelchair users in either the ballottement test or DRUJ instability assessed by ultrasonography. A survey of wheelchair users showed that a high proportion of participants had experienced severe TFCC injuries. Participants in the previous study indicated that these injuries were likely caused by overuse related to wheelchair driving or pushing up from a position of ulnar flexion in daily life [14]. The participants in the present study experienced repeated stress in their daily lives; however, the results suggest that the association between parasport participation and DRUJ instability was minimal. Although force-monitor ultrasonography has been validated in healthy volunteers, its reliability within this specific population has not yet been investigated. Therefore, further verification is needed to accurately interpret the results of this study.

Among participants with chronic spinal cord disorders, no significant differences were observed between the parasport and non-parasport groups regarding wrist ROM, upper limb muscle strength, hand function, upper limb circumference, and subjective upper limb-related difficulty as measured using the Q-DASH. In this study, the participants had thoracic or lumbar spinal cord disorders; therefore, they unlikely had actual spinal cord disorder-induced hand functional impairment. Research has previously recognized that the shoulder, wrist, fingers, and elbow, in that order, are the most frequently reported sites of secondary disorders impacting activities of daily living in wheelchair users with chronic spinal cord disorders [28]. The Q-DASH score showing a small number of participants with slight upper limb-related difficulties and wrist-related pain in daily life may be attributed to secondary disorders associated with activities of daily living, regardless of parasport participation. However, wrist loading related to activities of daily living and loading related to parasport participation were not analyzed in this pilot study and should be examined in future studies.

The most common disorders in non-athlete parasport competitors were reported to be those of the shoulder/upper arm, head/neck, hand/fingers, and elbow/forearm, in that order, due to contact and overuse [1]. The risk of upper limb disorders when parasports are introduced at the recreational level is expected due to unfamiliar contact and overuse in parasport-related movements. Research on shoulder disorders, the most common musculoskeletal issue affecting daily life—has reported that home exercise interventions can be effective in preventing the onset and exacerbation of shoulder pain [8]. No reports detail the effectiveness of preventive methods for wrist disorders; however, one report suggested the importance of wrist extension strength training in wheelchair basketball [29]. Wrist ROM has been reported to impact wheelchair basketball performance [30]. In this study, the participants may have had disorders and received medical interventions in conjunction with past parasport participation, but this aspect was not investigated. However, just as the sites of disorders vary depending on the sport, individualized approaches to managing overuse and injury prevention related to parasport participation—including for the wrist—are considered necessary according to the specific sport.

The present study included only one athlete-level participant, and the study findings showed no adverse impact of recreational parasport participation on hand function. Most parasport participation was for non-competitive recreational purposes (43.5%), and exercise was conducted 3.83 ± 1.97 days per week, with an average daily exercise time of 102 ± 58 min [31]. Parasport participation has recently been reported to be positively correlated with subjective well-being. Additionally, participation experience, rather than participation duration, was shown to have an impact on subjective well-being [32]. Recreational parasport participation is a purposeful activity option and may not be a decisive factor that increases the risk of upper limb joint disorders and functional impairments associated with competition. Prevention of disorders associated with parasport participation is essential to support continued engagement in parasports and to enhance subjective well-being.

### Study limitations and future implications

This study had some limitations. First, the statistical analysis was constrained by insufficient power due to the small sample size, highlighting the need for further research with a larger cohort to draw definitive conclusions. Additionally, as a cross-sectional pilot study, it could not establish a causal relationship between parasport participation and hand function. Future prospective longitudinal studies, informed by the findings of this pilot study, are necessary to evaluate the long-term effects of parasport participation on hand function. Second, while the present study focused on the evaluation of hand function, it did not consider subjective aspects and environmental differences, which are important for analyzing parasport participation. Support for promoting parasport participation includes the use of assistive devices, as well as team-based medical care involving individuals, families, and professionals, such as physicians, physical therapists, occupational therapists, psychologists, and physical educators [33, 34]. Promoting parasport participation that allows individuals to set their own goals and preventing disorders require approaches that address not only physical and mental functions but also psychological and environmental factors. This pilot study did not show any clear differences in hand function based on parasport participation. However, further research is needed to explore factors, such as sense of happiness and meaning associated with participation, wheelchair setup, welfare equipment used, and medical and welfare services. Third, the number of participants and sports in this pilot study was limited. In addition, the number of years of parasport experience for each participant was not considered. Consequently, the results do not reflect the participation status for all parasports. Further research involving wheelchair users with diverse athletic experiences is warranted. Fourth, the degree and frequency of mechanical stress on the wrist vary by parasport discipline. As this study included participants engaged in various disciplines, the specific effects of individual sports may have been masked, potentially introducing heterogeneity into the results. Therefore, caution is warranted when generalizing these findings. Future studies should incorporate subgroup analyses based on parasport discipline and intensity to better understand sport-specific risks. Fifth, this study primarily involved non-athletes participating at a recreational level. The mechanical demands on the hands likely differ between elite athletes and recreational participants, and as a result, the associated risks and required preventive strategies may differ. Finally, the presence of a TFCC injury could not be confirmed for all participants. The possibility of hidden TFCC injuries in both inexperienced and experienced parasport participants cannot be ruled out. However, caution should be exercised in interpreting the results because TFCC injuries are not always associated with DRUJ instability or clinical symptoms. In addition, the presence of a history of TFCC injury should be controlled and compared between groups.

## Conclusions

This pilot study preliminarily and exploratorily investigated the relationship between parasport participation and hand function in wheelchair users with chronic spinal cord injuries. Within this limited sample, no significant differences in DRUJ instability or hand function were observed between those who participated in recreational-level parasports and those who did not. Recreational parasport participation may not have an adverse impact on hand function in wheelchair users with chronic spinal cord disorders, provided that measures are taken to prevent contact and overuse in parasport-related movements. However, as this was a pilot cross-sectional study, it does not allow for conclusions concerning causality. Therefore, factors that may influence hand function should be further investigated in future research.

## Data Availability

The datasets generated and/or analyzed during the current study are not publicly available owing to ethical restrictions but are available from the corresponding author upon reasonable request.

## Abbreviations

DRUJ: Distal radioulnar joint
IQR: Interquartile range
Q-DASH: Quick disability of the arm, shoulder, and hand
ROM: Range of motion
TFCC: Triangular fibrocartilage complex

## References

[1] Osmotherly PG, Thompson E, Rivett DA, Haskins R, Snodgrass SJ. Injuries, practices and perceptions of Australian wheelchair sports participants. Disabil Health J. 2021;14:101044. 33248931 10.1016/j.dhjo.2020.101044

[2] Stephens C, Neil R, Smith P. The perceived benefits and barriers of sport in spinal cord injured individuals: a qualitative study. Disabil Rehabil. 2012;34:2061–70. 22494335 10.3109/09638288.2012.669020

[3] Luijten SCM, Te Loo LM, Nauta J, Janssen TWJ, Holla JFM, Otten RHJ, et al. Sports-related health problems in para-sports: a systematic review with quality assessment. Sports Health. 2024;16:551–64. 37337621 10.1177/19417381231178534 PMC11195855

[4] Derman W, Schwellnus MP, Jordaan E, Runciman P, Blauwet C, Webborn N, et al. Sport, sex and age increase risk of illness at the Rio 2016 summer paralympic games: a prospective cohort study of 51 198 athlete days. Br J Sports Med. 2018;52:17–23. 29074477 10.1136/bjsports-2017-097962

[5] Derman W, Schwellnus MP, Jordaan E, Runciman P, Van de Vliet P, Blauwet C, et al. The incidence and patterns of illness at the Sochi 2014 winter paralympic games: a prospective cohort study of 6564 athlete days. Br J Sports Med. 2016;50:1064–8. 27162232 10.1136/bjsports-2016-096215

[6] Willick SE, Webborn N, Emery C, Blauwet CA, Pit-Grosheide P, Stomphorst J, et al. The epidemiology of injuries at the London 2012 paralympic games. Br J Sports Med. 2013;47:426–32. 23515713 10.1136/bjsports-2013-092374

[7] Soo Hoo JA, Latzka E, Harrast MA. A descriptive study of self-reported injury in non-elite adaptive athletes. PM R. 2018. 10.1016/j.pmrj.2018.08.386. 30195706 10.1016/j.pmrj.2018.08.386

[8] García-Gómez S, Pérez-Tejero J, Hoozemans M, Barakat R. Effect of a home-based exercise program on shoulder pain and range of motion in elite wheelchair basketball players: a non-randomized controlled trial. Sports (Basel). 2019;7:180. 31344871 10.3390/sports7080180 PMC6723715

[9] Wilroy J, Hibberd E. Evaluation of a shoulder injury prevention program in wheelchair basketball. J Sport Rehabil. 2018;27:554–9. 29140190 10.1123/jsr.2017-0011

[10] Maarouf A, Norasteh AA, Daneshmandi H, Atri AE. The effect of a corrective exercise program based on scapula stability on scapular upward rotation and scapulohumeral rhythm in wheelchair basketball athletes with bilateral scapula rotational syndrome. Arch Rehabil. 2021;21:488–507.

[11] Demeco A, de Sire A, Marotta N, Palumbo A, Fragomeni G, Gramigna V, et al. Effectiveness of rehabilitation through kinematic analysis of upper limb functioning in wheelchair basketball athletes: a pilot study. Appl Sci. 2022;12:2929.

[12] Sakai M, Mutsuzaki H, Shimizu Y, Okamoto Y, Nakajima T. Characteristic MRI findings of the shoulder, elbow, and wrist joints in elite wheelchair basketball players. BMC Sports Sci Med Rehabil. 2022;14:141. 35870996 10.1186/s13102-022-00528-9 PMC9308260

[13] Yuine H, Mutsuzaki H, Yoshii Y, Shimizu Y, Ishida N, Yasuda T, et al. Evaluation of hand functions and distal Radioulnar joint instability in elite wheelchair basketball athletes: a cross-sectional pilot study. BMC Sports Sci Med Rehabil. 2023;15:58. 37061701 10.1186/s13102-023-00658-8 PMC10105936

[14] Sakai M, Mutsuzaki H, Shimizu Y, Okamoto Y, Yatabe K, Muraki I, et al. Correction to: Characteristic MRI findings of shoulder, elbow, and wrist joints in wheelchair user. Skelet Radiol. 2021;50:843. 10.1007/s00256-021-03717-8 33483773

[15] Andersson JK, Axelsson P, Strömberg J, Karlsson J, Fridén J. Patients with triangular fibrocartilage complex injuries and distal Radioulnar joint instability have reduced rotational torque in the forearm. J Hand Surg Eur Vol. 2016;41:732–8. 26701974 10.1177/1753193415622342

[16] Yoshii Y, Ishii T, Tung WL. Ultrasound assessment of the effectiveness of carpal tunnel release on median nerve deformation. J Orthop Res. 2015;33:726–30. 25664970 10.1002/jor.22843

[17] Yoshii Y, Yuine H, Tung WL, Ishii T. Quantitative assessment of distal Radioulnar joint stability with pressure-monitor ultrasonography. J Orthop Surg Res. 2019;14:195. 31248433 10.1186/s13018-019-1237-3 PMC6598296

[18] Yuine H, Yoshii Y, Tung WL, Ishii T, Shiraishi H. Reliability of quantitative assessment of distal Radioulnar joint stability with force-monitor ultrasonography. J Orthop Res. 2019;37:2053–60. 31062374 10.1002/jor.24331

[19] Imaeda T, Toh S, Wada T, Uchiyama S, Okinaga S, Kusunose K, et al. Validation of the Japanese society for surgery of the hand version of the quick disability of the Arm, Shoulder, and hand (QuickDASH-JSSH) questionnaire. J Orthop Sci. 2006;11:248–53. 16721524 10.1007/s00776-006-1013-1 PMC2778693

[20] Kunselman AR. A brief overview of pilot studies and their sample size justification. Fertil Steril. 2024;121:899–901. 38331310 10.1016/j.fertnstert.2024.01.040 PMC11128343

[21] Cohen J. A power primer. Psychol Bull. 1992;112:155–9. 19565683 10.1037//0033-2909.112.1.155

[22] Cooper H, Hedges LV, Valentine JC. *The Handbook of Research Synthesis and Meta-Analysis.* 2nd edition; 2009. pp. 231–234.

[23] Wijffels M, Brink P, Schipper I. Clinical and non-clinical aspects of distal Radioulnar joint instability. Open Orthop J. 2012;6:204–10. 22675411 10.2174/1874325001206010204 PMC3367466

[24] Rodríguez-Merchán EC, Shojaie B, Kachooei AR. Distal Radioulnar joint instability: diagnosis and treatment. Arch Bone Jt Surg. 2022;10:3–16. 35291239 10.22038/ABJS.2021.57194.2833 PMC8889419

[25] Yuine H, Yoshii Y, Iwai K, Ishii T, Shiraishi H. Application of force-monitor ultrasonography to assess distal Radioulnar joint instability in patients with triangular fibrocartilage complex injury. Ultrasound. 2022;30:219–27. 35936965 10.1177/1742271X211038351 PMC9354175

[26] Yuine H, Yoshii Y, Iwai K, Ishii T, Shiraishi H. Assessment of distal Radioulnar joint stability in healthy subjects: changes with dominant hand, sex, and age. J Orthop Res. 2021;39:2028–35. 33002205 10.1002/jor.24870

[27] Yuine H, Yoshii Y, Miyata K, Shiraishi H. Quantitative assessment of the course of distal Radioulnar joint instability. Hand Ther. 2022;27:83–90. 37905198 10.1177/17589983221113872 PMC10588430

[28] Dalyan M, Cardenas DD, Gerard B. Upper extremity pain after spinal cord injury. Spinal Cord. 1999;37:191–5. 10213328 10.1038/sj.sc.3100802

[29] Akınoğlu B, Kocahan T. Characteristics of upper extremity’s muscle strength in Turkish National wheelchair basketball players team. J Exerc Rehabil. 2017;13:62–7. 28349035 10.12965/jer.1732868.434 PMC5332001

[30] Wang YT, Chen S, Limroongreungrat W, Change L-S. Contributions of selected fundamental factors to wheelchair basketball performance. Med Sci Sports Exerc. 2005;37:130–7. 15632679 10.1249/01.mss.0000150076.36706.b2

[31] Carlin L, McPherson G, Davison R. The international classification of functioning disability and health framework (ICF): a new approach to enhance sport and physical activity participation among people with disabilities in Scotland. Front Sports Act Living. 2024;6:1225198. 38558859 10.3389/fspor.2024.1225198 PMC10978736

[32] Martin Ginis KA, Gee CM, Sinden AR, Tomasone JR, Latimer-Cheung AE. Relationships between sport and exercise participation and subjective well-being among adults with physical disabilities: is participation quality more important than participation quantity? Psychol Sport Exerc. 2024;70:102535. 37696314 10.1016/j.psychsport.2023.102535

[33] Geppert A, Smith EM, Haslett D, Wong J, Ebuenyi ID, MacLachlan M. Assistive technology to promote participation in sport for people with disabilities. Stud Health Technol Inf. 2023;306:191–8. 10.3233/SHTI230618 37638915

[34] Clutterbuck GL, Sousa Junior RR, Leite HR, Johnston LM. The SPORTS participation framework: illuminating the pathway for people with disability to enter into, participate in, and excel at sport. Braz J Phys Ther. 2024;28:101081. 38851054 10.1016/j.bjpt.2024.101081 PMC11208908

